# Antibacterial Activity and Mechanism of Action of Sulfone Derivatives Containing 1,3,4-Oxadiazole Moieties on Rice Bacterial Leaf Blight

**DOI:** 10.3390/molecules200711660

**Published:** 2015-06-24

**Authors:** Li Shi, Pei Li, Wenli Wang, Manni Gao, Zengxue Wu, Xianpeng Song, Deyu Hu

**Affiliations:** State Key Laboratory Breeding Base of Green Pesticide and Agricultural Bioengineering, Key Laboratory of Green Pesticide and Agricultural Bioengineering, Ministry of Education, Guizhou University, Huaxi District, Guiyang 550025, China; E-Mails: s2012l@sina.com (L.S.); pl19890627@126.com (P.L.); wangwenli0208@163.com (W.W.); gaodunanqi@126.com (M.G.); wzx3120@163.com (Z.W.); sxp1529@163.com (X.S.)

**Keywords:** sulfone derivative, antibacterial activity, action mechanism, *Xanthomonas oryzae pv. oryzae*, extracellular polysaccharide

## Abstract

In this study, sulfone derivatives containing 1,3,4-oxadiazole moieties indicated good antibacterial activities against rice bacterial leaf blight caused by the pathogen *Xanthomonas* *oryzaepv.* *pv. oryzae* (*Xoo*). In particular, 2-(methylsulfonyl)-5-(4-fluorobenzyl)-1,3,4-oxadiazole revealed the best antibacterial activity against *Xoo*, with a half-maximal effective concentration (EC_50_) of 9.89 μg/mL, which was better than those of the commercial agents of bismerthiazole (92.61 μg/mL) and thiodiazole copper (121.82 μg/mL). *In vivo* antibacterial activity tests under greenhouse conditions and field trials demonstrated that 2-(methylsulfonyl)-5-(4-fluorophenyl)-1,3,4-oxadiazole was effective in reducing rice bacterial leaf blight. Meanwhile, 2-(methylsulfonyl)-5-(4-fluorophenyl)-1,3,4-oxadiazole stimulate the increase in superoxide dismutase (SOD) and peroxidase (POD) activities in rice, causing marked enhancement of plant resistance against rice bacterial leaf blight. It could also improve the chlorophyll content and restrain the increase in the malondialdehyde (MDA) content in rice to considerably reduce the amount of damage caused by *Xoo*. Moreover, 2-(methylsulfonyl)-5-(4-fluorophenyl)-1,3,4-oxadiazole, at a concentration of 20 μg/mL, could inhibit the production of extracellular polysaccharide (EPS) with an inhibition ratio of 94.52%, and reduce the gene expression levels of *gumB*, *gumG*, *gumM*, and *xanA*, with inhibition ratios of 94.88%, 68.14%, 86.76%, and 79.21%, respectively.

## 1. Introduction

Rice is one of the most important staple crops around the world. Unfortunately, grain yield has decreased significantly because of rice bacterial leaf blight, which is caused by the pathogen *Xanthomonas* *oryzae* *pv. oryzae* (*Xoo*), the most important and well-known bacterial disease of rice in rice-growing regions. Bacterial leaf blight can cause leaf wilting, affect photosynthesis, reduce 1000-grain weight, and generally result in yield losses by 20%–30% and even 100% under severe conditions [1,2,3,4,5]. Although bismerthiazole and streptomycin are the main tools for controlling rice bacterial leaf blight in China, *Xoo* has developed high resistance to both these bactericides [6,7]. Therefore, the search for new antibacterial agents remains a difficult task, and such agents are greatly needed in the field of agricultural bactericides.

**Figure 1:**
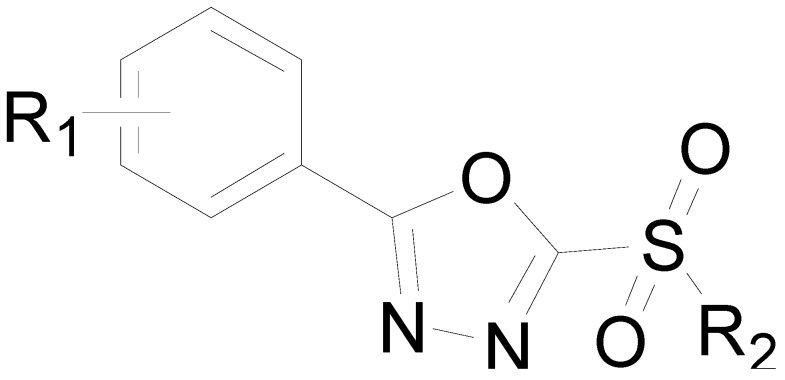
Compounds previously reported against tobacco and tomato bacterial wilts.

Sulfone derivatives containing 1,3,4-oxadiazole moieties have a broad spectrum of bioactivities, such as antibacterial [8,9,10], antifungal [11,12], insecticidal [13], herbicidal [14], anticancer [15], and anti-HIV-1 [16] properties. Over the past few years, studies on the synthesis and bioactivity of sulfone derivatives containing 1,3,4-oxadiazole moieties have attracted considerable attention. We previously demonstrated that such sulfone derivatives (Figure 1) display potent antibacterial activities. Specifically, 2-(methylsulfonyl)-5-(4-fluorophenyl)-1,3,4-oxadiazole (CAS Registry Number: 142225-95-4) showed the best antibacterial activity against tobacco and tomato bacterial wilts caused by *Ralstonia* *solanacearum* (*R.* *solanacearum*) with half-maximal effective concentration (EC_50_) values of 8.29 and 19.77 μg/mL, respectively [8]. However, in our previous work, we only reported and discussed the compound’s activities in the control of *R. solanacearum*. The biological effects of these sulfone derivatives containing 1,3,4-oxadiazole moieties against rice bacterial leaf blight were not reported, and the underlying mechanism of these compounds on rice bacterial leaf blight remained unclear.

In this study, we found that 1,3,4-oxadiazole-containg sulfone derivatives, which demonstrate potent antibacterial activities against *R. solanacearum*, were highly effective against rice bacterial leaf blight *in vitro* and *in vivo*. Meanwhile, 2-(methylsulfonyl)-5-(4-fluorophenyl)-1,3,4-oxadiazole could stimulate an increase in superoxide dismutase (SOD) and peroxidase (POD) activities in rice, causing a marked enhancement of plant resistance against rice bacterial leaf blight. It could also improve the chlorophyll content and restrain the increase in the malondialdehyde (MDA) content in rice to considerably reduce the amount of damage caused by *Xoo*. Moreover, 2-(methylsulfonyl)-5-(4-fluorophenyl)-1,3,4-oxadiazole could obviously inhibit the production of extracellular polysaccharide (EPS) and reduce the gene expression levels of *gumB*, *gumG*, *gumM*, and *xanA*.

## 2. Results and Discussion

### 2.1. In Vitro Antibacterial Bioassay

As shown in Table 1, all the tested compounds demonstrated potent antibacterial activities against *Xoo*, with half-maximal effective concentration (EC_50_) values ranging from 9.89 μg/mL to 63.59 μg/mL, which were even better than those of bismerthiazole (92.61 μg/mL) and thiodiazole copper (121.82 μg/mL). The antibacterial tests showed that when small electron-with-drawing groups (e.g., -F) at the 4-position and a methyl substituted sulfonyl substituent were attached to the oxadiazole 2,5-positions, the corresponding compound 2-(methylsulfonyl)-5-(4-fluorophenyl)-1,3,4-oxadiazole presented the best antibacterial activity compared to the rest of the test compounds. Meanwhile, the *in vitro* activity against *Xoo* of the compound 2-(methylsulfonyl)-5-(4-fluorophenyl)-1,3,4-oxadiazole, with an EC_50_ value of 9.89 μg/mL, was better than the activity against tomato bacterial wilt (19.77 μg/mL) and slightly below the activity against tobacco bacterial wilt (8.29 μg/mL).

**Table 1:** Antibacterial activities against *Xanthomonas* *oryzae* *pv. oryzae*of the title compounds.

Compds.	Toxic Regression Equation	*R*	EC_50_ (μg/mL)
2-(Methylsulfonyl)-5-phenyl-1,3,4-oxadiazole	y = 2.16x + 2.18	0.98	20.07 ± 1.21
2-(Ethylsulfonyl)-5-phenyl-1,3,4-oxadiazole	y = 1.52x + 2.77	0.98	29.00 ± 1.25
2-(Methylsulfonyl)-5-(4-fluorophenyl)-1,3,4-oxadiazole	y = 4.13x + 0.89	0.95	9.89 ± 1.52
2-(Ethylsulfonyl)-5-(4-fluorophenyl)-1,3,4-oxadiazole	y = 3.28x + 1.61	0.96	10.80 ± 1.43
2-(Methylsulfonyl)-5-(4-chlorophenyl)-1,3,4-oxadiazole	y = 1.72x + 2.64	0.99	23.21 ± 0.98
2-(Ethylsulfonyl)-5-(4-chlorophenyl)-1,3,4-oxadiazole	y = 1.60x + 2.25	0.99	52.61 ± 1.08
2-(Methylsulfonyl)-5-(2,4-dichlorophenyl)-1,3,4-oxadiazole	y = 1.04x + 3.21	0.99	52.14 ± 1.05
2-(Ethylsulfonyl)-5-(2,4-dichlorophenyl)-1,3,4-oxadiazole	y = 1.43x + 2.42	0.97	63.95 ± 1.05
Bismerthiazole	y = 1.50x + 2.05	0.98	92.61 ± 2.15
Thiodiazole copper	y = 1.54x + 1.79	0.98	121.82 ± 3.59

### 2.2. In Vivo Antibacterial Bioassay

The results are listed in Table 2. At 15th day after spraying, 2-(methylsulfonyl)-5-(4-fluorophenyl)-1,3,4-oxadiazole had potent curative activity of 38.17% against rice bacterial leaf blight at 200 μg/mL, which was better than those of bismerthiazole (30.21%) and thiodiazole copper (29.51%). At 28th day after spraying, the protective activity of 2-(methylsulfonyl)-5-(4-fluorophenyl)-1,3,4-oxadiazole against rice bacterial leaf blight at 200 μg/mL was 41.82%, which was superior to those of bismerthiazole (37.51%) and thiodiazole copper (25.58%).

**Table 2:** Control efficiency of the testing compounds against rice bacterial leaf blight under greenhouse conditions at the concentration of 200 μg/mL.

Compds.	15 Days after Spraying		28 Days after Spraying	
	Disease Index (%)	Curative Activity (%) ^c^	Disease Index (%)	Protection Activity (%) ^c^
2-(Methylsulfonyl)-5-(4-fluorophenyl)-1,3,4-oxadiazole	57.63 ± 3.51	38.17 ± 2.15 ^A^	54.79 ± 2.78	41.82 ± 2.45 ^A^
Bismerthiazole ^a^	65.05 ± 2.26	30.21 ± 3.43 ^B^	58.85 ± 3.12	37.51 ± 2.54 ^C^
Thiodiazole copper ^b^	65.70 ± 2.73	29.51 ± 4.76 ^C^	70.08 ± 3.67	25.58 ± 2.42 ^B^
Untreated blank control	93.21 ± 1.79	0	94.17 ± 2.55	0

^a^: 20% WP; ^b^: 3% WP; ^c^: Statistical analysis was conducted via the ANOVA method at a condition of equal variances assumed (*p* > 0.05) and equal variances not assumed (*p* < 0.05). Different capital letters indicate the values of control efficiency with significant difference among different treatment groups at *p* < 0.05.

### 2.3. Field Trial against Rice Bacterial Leaf Blight

The results are summarized in Table 3. At 15th day after the third spraying, the control efficiency of 2-(methylsulfonyl)-5-(4-fluorophenyl)-1,3,4-oxadiazole at 150 g ai/ha against rice bacterial leaf blight was 81.34%, which was better than those of bismerthiazole (76.19%) and zhongshengmycin (71.87%).

**Table 3:** Field trials of the testing compounds against rice bacterial leaf blight.

Compds.	Dosage (g ai/ha) ^d^	15 Days after the Third Spraying	
		Disease Index (%)	Control Efficiency (%) ^e^
2-(Methylsulfonyl)-5-(4-fluorophenyl)-1,3,4-oxadiazole ^a^	150	2.03 ± 1.22	81.34 ± 2.76 ^A^
Bismerthiazole ^b^	375	2.59 ± 1.54	76.19 ± 3.54 ^C^
Zhongshengmycin ^c^	45	3.06 ± 1.86	71.87 ± 4.33 ^B^
Untreated blank control	0	10.88 ± 2.32	0

^a^: 20% SC; ^b^:20% WP; ^c^: 3% WP; ^d^: Active ingredient; ^e^: Statistical analysis was conducted via the ANOVA method at a condition of equal variances assumed (*p* > 0.05) and equal variances not assumed (*p* < 0.05). Different capital letters indicate the values of control efficiency with significant difference among different treatment groups at *p* < 0.05.

### 2.4. Determination of SOD and POD Activities

SOD, a key enzyme that resists biological oxidation in plant, catalyzes the reduction of superoxide anions (O_2_^−^) to hydrogen peroxide (H_2_O_2_). The diminished capacity for O_2_^−^ removal causes a decreased ability of progeria cells to minimize oxidative damage may be a key factor in the disease. It plays a critical role in the defense of cells against the toxic effects of oxygen radicals [17].

POD constitutes a class of enzymes extensively distributed in plants and it has been shown that POD plays an active role in metabolism. An important function attributed to POD in plants concerns lignin synthesis. In many cases, particularly for plant-microbe interactions, this has been suggested as defense responses of plants to the stress [18].

**Figure 2:**
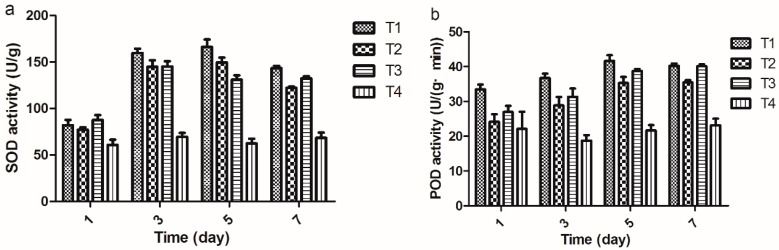
The changes of SOD (**a**) and POD (**b**) activities (The data presented are the mean ± SD). **T1**: *Xoo* + Compound 2-(methyl sulfonyl)-5-(4-fluorophenyl)-1,3,4-oxadiazole; **T2**: *Xoo* + Bismerthiazol; **T3**: *Xoo*; **T4**: Untreated blank control.

As shown in Figure 2, SOD and POD activities in rice were enhanced by 2-(methylsulfonyl)-5-(4-fluorophenyl)-1,3,4-oxadiazole at all sampling times, and the contents changed approximately in a Λ-shape manner and peaked on the 5th day with a rate of increase of 62.67% and 50.65%, respectively. Nevertheless, SOD and POD activities showed a declining tendency as time progressed from 5th day to 7th day. The value during this period was observed to be higher than the one inoculated by *Xoo* and treated with water. These results showed that 2-(methylsulfonyl)-5-(4-fluorophenyl)-1,3,4-oxadiazolecould improve the disease resistance of plants, which rely on inducible defense responses in the form of enzymes that are activated for controlling rice bacterial leaf blight. 

### 2.5. Determination of Chlorophyll Content in Rice

Photosynthesis is a special, and the most basic, life process of green plants providing themselves necessary growth and energy [19]. Chlorophyll is the photosynthetic organelle of green plants whose content is closely related to photosynthesis, extent of bacterial infection in plants leading to proliferation and destruction of the plant chloroplasts and factors retarding the synthesis of chlorophyll causing leaf chlorosis [20].

**Figure 3:**
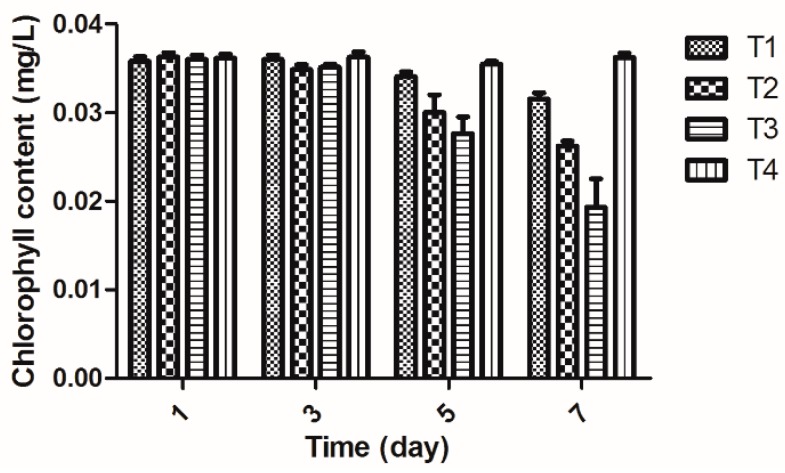
The changes of chlorophyll content in rice (The data presented are the mean ± SD). **T1**: *Xoo* + Compound 2-(methylsulfonyl)-5-(4-fluorophenyl)-1,3,4-oxadiazole; **T2**: *Xoo* + Bismerthiazole; **T3**: *Xoo*; **T4**: Untreated blank control.

As shown in Figure 3, after rice leaves were inoculated with *Xoo*, the chlorophyll content decreased during days 1–7 and reached the lowest value on the 7th day. However, the chlorophyll content of rice leaves treated with 2-(methylsulfonyl)-5-(4-fluorophenyl)-1,3,4-oxadiazole was higher than those of leaves inoculated with *Xoo* and treated by bismerthiazole, but lower than those of leaves treated with water during days 1–7. Figure 3 also shows that *Xoo* infection of rice lowered the chlorophyll content, but rice treated by 2-(methylsulfonyl)-5-(4-fluorophenyl)-1,3,4-oxadiazole showed an enhancement in chlorophyll content. This finding indicated that 2-(methylsulfonyl)-5-(4-fluorophenyl)-1,3,4-oxadiazole may destroy *Xoo* in rice, thereby enhancing the host’s resistance to disease. However, the chlorophyll content of rice treated with 2-(methylsulfonyl)-5-(4-fluorophenyl)-1,3,4-oxadiazole in each treatment period was found to be lower than that in the healthy control, indicating that 2-(methyl- sulfonyl)-5-(4-fluorophenyl)-1,3,4-oxadiazole may not completely suppress *Xoo*-induced chloroplast damage.

### 2.6. Determination of MDA Content in Rice

Increasing appreciation of the causative role of oxidative injury in many disease states places great importance on the reliable assessment of lipid peroxidation. MDA is one of several low-molecular-weight end products formed via the decomposition of certain primary and secondary lipid peroxidation products [21].

**Figure 4:**
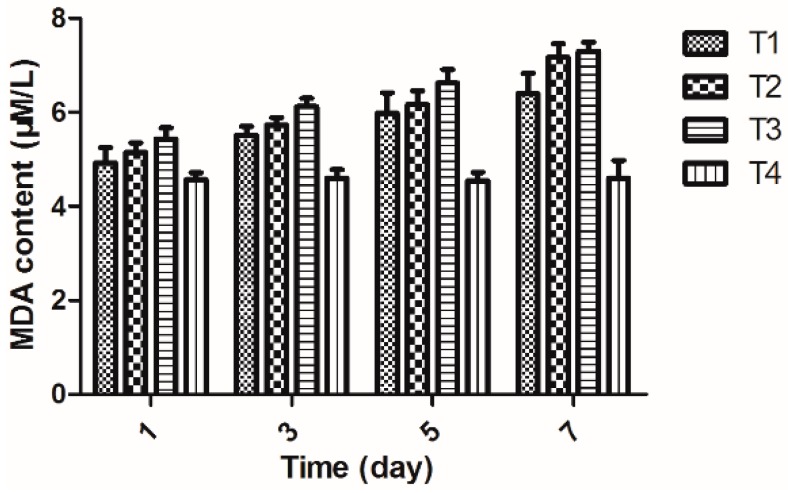
The changes of MDA content in rice (The data presented are the mean ± SD). **T1**: *Xoo* + Compound 2-(methyl sulfonyl)-5-(4-fluorophenyl)-1,3,4-oxadiazole; **T2**: *Xoo* + Bismerthiazol; **T3**: *Xoo*; **T4**: Untreated blank control.

As shown in Figure 4, the MDA content in rice was increased in different treatment groups at all sampling times. It reached the highest value at 7th day, and it increased to 6.39 μM/L after treatment with 2-(methylsulfonyl)-5-(4-fluorophenyl)-1,3,4-oxadiazole. This value was higher than that in rice treated with water (6.39 μM/L) but lower than the one inoculated with *Xoo* (7.30 μM/L) and the one treated with bismerthiazole (7.17 μM/L) during 1–7 days. The MDA content demonstrated that 2-(methylsulfonyl)-5-(4-fluorophenyl)-1,3,4-oxadiazole could restrain the increase in the MDA content in rice, thereby enhancing the host’s resistance to the disease.

### 2.7. Biofilm Formation

**Figure 5:**
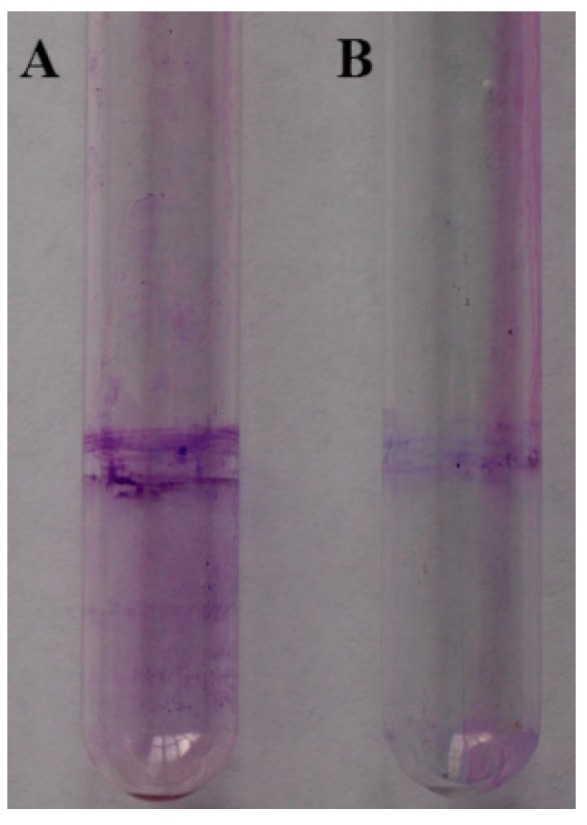
Effect on the biofilm formation. **A**: Untreated blank control; **B**: *Xoo* + Compound 2-(methylsulfonyl)-5-(4-fluorophenyl)-1,3,4-oxadiazole.

2-(Methylsulfonyl)-5-(4-fluorophenyl)-1,3,4-oxadiazole was hypothesized to be involved in biofilm formation. As shown in Figure 5, less biofilm was observed for the treatment of 2-(methylsulfonyl)-5-(4-fluorophenyl)-1,3,4-oxadiazole than the blank control. The absorbance of crystal violet in the biofilm-staining assay for the blank control (optical density at 570 nm (OD_570_), 0.300) was 1.5 times greater than that of the treatment group (OD_570_, 0.204).Three replicates were used for quantitative measurements of biofilm.

### 2.8. Quantitative Determination of EPS Production

EPS, high-molecular weight compounds secreted by microorganisms into their environment, establish the functional and structural integrity of biofilms. EPS are considered the fundamental component that determines the physiochemical properties of a biofilm. EPS can protect pathogenic bacteria and contribute to their pathogenicity. A previous study reported that EPS, one pathogenic factor of *Xoo*, can lead to wilting of rice leaves and reduce EPS production to decrease the pathogenicity.

**Figure 6:**
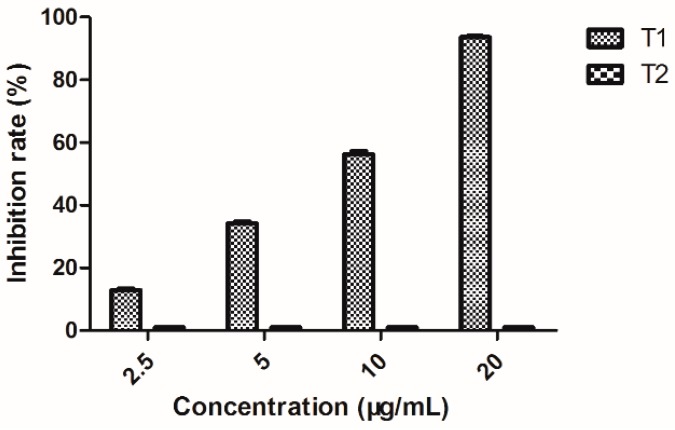
Inhibition rate of EPS of *Xoo* (The data presented are the mean ± SD). **T1**: *Xoo* + Compound 2-(methylsulfonyl)-5-(4-fluorophenyl)-1,3,4-oxadiazole; **T2**: Untreated blank control.

As shown in Figure 6, 2-(methylsulfonyl)-5-(4-fluorophenyl)-1,3,4-oxadiazole, at the concentrations of 2.5, 5, 10, and 20 μg/mL, could obviously inhibit the EPS production of *Xoo*, with inhibition rates of 12.76%, 34.29%, 56.47%, and 94.52%, respectively. These results demonstrated that 2-(methylsulfonyl)-5-(4-fluorophenyl)-1,3,4-oxadiazolecould reduce EPS production to lower the pathogenic ability of *Xoo*.

### 2.9. EPS Gene Expression Level in Xoo

The biosynthetic pathway by which EPS is produced involves three steps: the first step is the conversion of simple sugars to nucleotidyl derivative precursors; the second step is the assembly of pentasaccharide subunits attached to an inner-membrane polyprenol phosphate carrier, with addition of acetyl and pyruvate groups; and the third step is the polymerization of the pentasaccharide repeating units and secretion of EPS [22]. *XanA* coding for phosphoglucomutase/phosphomannomutase, which can be glucose-6-phosphate into glucose-1-phosphate in the first step of EPS biosynthesis [23]. The genes encoding the proteins related to the last two steps of EPS biosynthesis are encoded by the gum gene cluster [24]. GumM (the protein of *gumM*) plays an important role in the process of the biosynthesis of the pentasaccharide, GumG (the protein of *gumG*) is partly responsible for modification of the pentasaccharide, and GumB (the protein of *gumB*) is a key role in the process of EPS polymerization and transport [25].

**Figure 7:**
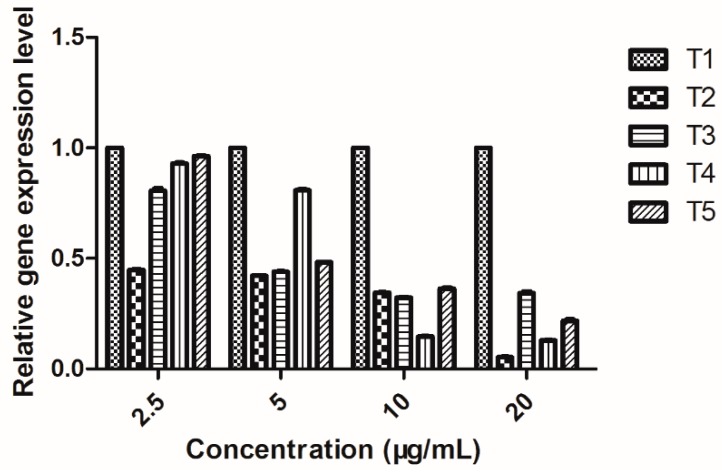
Expression of EPS gene of *Xoo* (The data presented are the mean ± SD). **T1**: Untreated blank control; **T2**: *gumB*; **T3**: *gumG*; **T4**: *gumM*; **T5**: *xanA*.

In this study, the expression levels of four EPS genes (*gumB*, gumG, *gumM*, and *xanA*) in *Xoo* were determined by RT-qPCR. As evident from Figure 7, all four genes of *Xoo* treated with 2-(methyl- sulfonyl)-5-(4-fluorophenyl)-1,3,4-oxadiazole, showed lower expression levels than the untreated blank control. Meanwhile, Table 4 shows that the expression of *xanA* was lowest at 20 μg/mL, with the inhibition rate of 79.21%, which implied that compound 2-(methylsulfonyl)-5-(4-fluorophenyl)-1,3,4-oxadiazole may hindered the biosynthesis of phosphoglucomutase/phosphomannomutase in *Xoo*, and then reduced glucose-6-phosphate into glucose-1-phosphate in the first step of EPS biosynthesis. 

The expression of *gumM* was lowest at 20 μg/mL, with the inhibition rate of 86.76%, which suggested that compound 2-(methylsulfonyl)-5-(4-fluorophenyl)-1,3,4-oxadiazole may impeded the biosynthesis of the pentasaccharide of *Xoo*. Meanwhile, Table 4 showed that the expression of *gumG* was lowest at 20 μg/mL, with the inhibition rate of 68.14%, which suggested that compound 2-(methylsulfonyl)-5-(4-fluorophenyl)-1,3,4-oxadiazole possibly receded the modification of the pentasaccharide of *Xoo*. The expression of *gumB* was lowest at 20 μg/mL, with the inhibition rate of 94.88%, which meant that compound 2-(methylsulfonyl)-5-(4-fluorophenyl)-1,3,4-oxadiazole could destroy the process of EPS polymerization and transport of *Xoo*.

**Table 4:** Inhibition rates of 2-(methylsulfonyl)-5-(4-fluorophenyl)-1,3,4-oxadiazole on EPS gene expression at 20 μg/mL.

Genes	Inhibition Rate (%)
*gumB*	94.88 ± 0.39
*gumG*	68.14 ± 1.15
*gumM*	86.76 ± 0.28
*xanA*	79.21 ± 1.14

## 3. Experimental Section

### 3.1. Bacterial Strains and Culture Conditions

PXO99A strain of *Xoo* was grown at 28 ± 1 °C in nutrient broth (NB) medium in conical flasks or on nutrient agar (NA) medium in Petri dishes [6]. NA medium was prepared with 1 g of yeast extract, 3 g of beef extract, 5 g of polypeptone, 10 g of sucrose, and 15 g of agar powder per 1000 mL of distilled water, pH 7.0–7.2. NB medium contained the same components but lacked agar powder.

### 3.2. In Vitro Antibacterial Activity

In this study, eight sulfone derivatives containing 1,3,4-oxadiazole moieties were evaluated for their antibacterial activities against *Xoo* via the turbidimeter test [26] *in vitro*. Dimethylsulfoxide (DMSO) in sterile distilled water served as an untreated blank control. Bismerthiazole and thiodiazole copper which are the principal tools used for controlling rice bacterial leaf blight in China at present served as positive controls,. Approximately 40 μL of solvent NB containing *Xoo*, incubated on the phase of logarithmic growth, was added to 5 mL of solvent NB containing the test compounds. The inoculated test tubes were incubated at 28 °C and continuously shaken at 180 rpm for 24–48 h until the bacteria were incubated on the logarithmic growth phase. The growth of the cultures was monitored on a Model 680 microplate reader (BIO-RAD, Hercules, CA, USA) by measuring the optical density at 595 nm (OD_595_). The inhibition rate *I* was calculated by the following formula:

Inhibition rate *I* (%) = (C − T)/C × 100 (1)

where C is the corrected turbidity values of bacterial growth on untreated NB (blank control), T is the corrected turbidity values of bacterial growth on treated NB, and *I* represents the inhibition rate.

Based on previous bioassays, the results of antibacterial activities (expressed by EC_50_) of the title compounds against *Xoo* were also evaluated and calculated with SPSS 17.0 software. The experiment was repeated three times.

### 3.3. In Vivo Antibacterial Activity

To determine the control efficiency of antibacterial potency *in vivo* at the greenhouse condition, the curative activity of 2-(methylsulfonyl)-5-(4-fluorophenyl)-1,3,4-oxadiazole against rice bacterial leaf blight was analyzed in potted plants using a complete randomized block design [27]. Seeds of Nipponbare were sowed in plastic pots that contained field soil and thinned to six to ten rice seedlings. *Xoo*, the pathogen of the rice disease of bacterial leaf blight, was cultured in solvent NB at 28 ± 1 °C overnight at 180 rpm, and the concentrations were then adjusted to 10^8^ CFU/mL. Five weeks after sowing, *Xoo* of rice bacterial leaf blight was inoculated on the rice plant. We used scissors, which were sterilized using 70% ethanol prior to use and then dipped in bacterial solution, to inoculate the rice plant with *Xoo*. After 7 d of *Xoo* inoculation, 200 μg/mL 2-(methylsulfonyl)-5-(4-fluorophenyl)-1,3,4-oxadiazole solution was sprayed until run-off onto the leaves, whereas the control plants were sprayed with the same volume of distilled water. All inoculated plants were incubated in a growth chamber at 28 °C and 90% relative humidity. At 15th day after inoculation, the average lesion length was observed and scored, and the control efficiency was calculated. The experiment was repeated three times.

Meanwhile, the protective activity of 2-(methylsulfonyl)-5-(4-fluorophenyl)-1,3,4-oxadiazole against rice bacterial leaf blight was also analyzed under greenhouse conditions. Five weeks after sowing the rice plant, 200 μg/mL 2-(methylsulfonyl)-5-(4-fluorophenyl)-1,3,4-oxadiazole solution was sprayed onto the leaves until run-off, whereas the control plants were sprayed with the same volume of distilled water. Seven d after 2-(methylsulfonyl)-5-(4-fluorophenyl)-1,3,4-oxadiazole spraying, *Xoo* rice bacterial leaf blight was inoculated on the rice plant. All inoculated plants were incubated in a growth chamber at 28 °C and 90% relative humidity. At 28th day after inoculation, the average lesion length was observed and scored, and the experiment was repeated three times. The control efficiency was calculated as follows:

Control efficiency *I* (%) = (C − T)/C × 100 (2)

where C represents the disease incidence of the untreated blank control, T represents the disease incidence of the treatment, and *I* represents the control efficiency.

### 3.4. Field Trial against Rice Bacterial Leaf Blight

To further determine the activities of 2-(methylsulfonyl)-5-(4-fluorophenyl)-1,3,4-oxadiazole, field trials against rice bacterial leaf blight were conducted. The effect of the natural infection of *Xoo* was studied in a field with rice having suffered rice bacterial leaf blight for several years. Sterile distilled water served as an untreated blank control, whereas the commercial bactericides bismerthiazole and zhongshengmycin were the positive controls. 2-(Methylsulfonyl)-5-(4-fluorophenyl)-1,3,4-oxadiazole (150 g ai/ha) and the commercial bactericides bismerthiazole (375 g ai/ha) and zhongshengmycin solutions (45 g ai/ha) were sprayed on the foliage of the rice once every 7 days three times. For each treatment, three replicates were conducted. The disease incidence of the rice plants was investigated 15th day after the third spraying. The control efficiency was calculated using the following formula:

Control efficiency *I* (%) = (C − T)/C × 100 (3)

where C represents the disease incidence of the untreated blank control, T represents the disease incidence of the treatment, and *I* represents the control efficiency.

### 3.5. Determination of SOD Activity

Rice samples (0.5 g) were homogenized in 0.05 M phosphate buffer (5 mL, pH 7.8) and centrifuged at 4000 *g* for 10 min. The supernatant was used as an enzyme source. The reaction mixture (3 mL) contained phosphate buffer (1.5 mL, 0.05 M, pH 7.8), methionine (0.3 mL, 130 mM), nitroblue tetrazolium (0.3 mL, 750 μM), ethylene diaminetetraaceticacid (EDTA) (0.3 mL, 100 μM), and riboflavin (0.3 mL, 20 μM). Phosphate buffer instead of enzyme liquid was set as blank control. The mixture was illuminated under a fluorescent lamp (4000 LUX, Ningbo Jiangnan Instrument Plant, Ningbo, China) for 20 min, and the absorbance was read at 560 nm. For the blank, identical solutions were kept under the dark. SOD activity was expressed as the change in absorbance g^−1^ fresh tissue [28].

### 3.6. Determination of POD Activity

Rice samples (1 g) were homogenized in 20 mM KH_2_PO_4_ (5 mL) and then centrifuged at 4000 *g* for 15 min at 4 °C. The supernatant was used as an enzyme source. The mixed reaction solution of POD consisted of 0.1 M phosphate buffer (500 mL, pH 6.0), guaiacol (280 μL), 30% H_2_O_2_ (190 μL) and 20 mM KH_2_PO_4_. To initiate the reaction, mixed reaction solution of POD (3 mL) and enzyme solution (0.1 mL) were added to the sample cuvette. Mixed reaction solution of POD and KH_2_PO_4_ were set as blank control. The absorbance was read at 470 nm about once a minute. POD activity was expressed as U·g^−1^ FW min^−1^ [29].

### 3.7. Determination of Chlorophyll Content

According to the work conducted by Peng [30], rice samples (10 mg) were homogenized in a 5 mL mixture of acetone and ethanol with a volume ratio of 4:1. Dark extraction was carried out for 1 h, and the extract was centrifuged at 4000 *g* for 5 min. The chlorophyll extract was then placed in a 1 cm-thick cuvette, a mixture of acetone and ethanol with a volume ratio of 4:1 was used as a reference. The absorbance was read at 645 and 663 nm:

C_a_ (mg·L^−1^) = 0.0127 A_663_ − 0.00269 A_645_ (4)

C_b_ (mg*·*L^−^^1^) = 0.0229A_645_ − 0.00468 A_663_ (5)

Total chlorophyll content = C_a_ + C_b_ (6)

In the above equation, A_645_ and A_663_ are the absorbance values at the wavelengths 645 and 663 nm, respectively.

### 3.8. Determination of MDA Content 

Rice samples (0.5 g) were homogenized in 5% trichloroacetic acid (TCA) (5 mL) and then centrifuged at 3000 *g* for 10 min. Subsequently, 0.67% barbituric acid (TBA) (2 mL) was added to the supernatant (2 mL). The mixture (4 mL) was boiled for 30 min at 100 °C and then centrifuged at 3000 g for 10 min. The absorbance was read at 450, 532, and 600 nm. The MDA content was expressed as C (μM*·*L^−1^):

C (μM*·*L^−^^1^) = 6.45(A_532_ − A_600_) − 0.56A_450_ [31] (7)

### 3.9. Biofilm Assays

Biofilm formation in glass test tube was quantified as described previously [32,33]. Bacteria were grown in NB with shaking to the mid-exponential growth phase and then diluted to 1:100 in fresh NB. About 2 mL of a diluted bacterial suspension was placed in each glass tube, incubated with 10 μg/mL 2-(methylsulfonyl)-5-(4-fluorophenyl)-1,3,4-oxadiazole, and shaken at 28 °C for 72 h. The culture medium was poured out, and attached bacterial cells were gently washed three times with distilled water. The cells were then stained with 0.1% crystal violet (2 mL) for 15 min. Unbound crystal violet was poured out, and the glass tubes were washed three times with water. The crystal violet-stained cells were solubilized in DMSO (2 mL). Biofilm formation was quantified by measuring the absorbance at 570 nm using a Synergy H1 detector (BioTek, Winooski, VT, USA). Three replicates were used for quantitative measurement.

### 3.10. Quantitative Determination of EPS Production 

To measure the influence of EPS production of *Xoo* in culture supernatants, bacterial cells were grown in NB supplemented with different concentrations (20, 10, 5, and 2.5 μg/mL) of 2-(methyl- sulfonyl)-5-(4-fluorophenyl)-1,3,4-oxadiazole at 28 °C for 72 h. Subsequently, 10 mL portions of the cultures were collected, and the cells were removed by centrifugation at 8000 g for 20 min [34]. Finally, three volumes of ethyl alcohol were added to the supernatants [35]. The precipitated EPS were pelleted via centrifugation, dried, and weighed. The test was performed three times independently.

### 3.11. RNA Extraction, cDNA Synthesis, and RT-qPCR Analysis

Bacteria were grown in NB medium at 28 °C with shaking at 180 rpm, and 1 mL samples of *Xoo* strain cultures were collected at 12 h after bacterial cells were incubated with different concentrations (20, 10, 5, and 2.5 μg/mL) of 2-(methylsulfonyl)-5-(4-fluorophenyl)-1,3,4-oxadiazole. Bacterial cells were centrifuged at 12,000 *g* for 10 min, and the cell pellets were treated with a Trizol reagent kit (TaKaRa, Dalian, China) [36]. Total RNA purity was estimated by calculating OD_260_/OD_280_ using an ultraviolet spectrophotometer (ACTGene, Piscataway, NJ, USA). All OD_260_/OD_280_ values of RNA were between 1.8 and 2.2. The concentration of total RNA was calculated according to the dilution ratio and OD_260_.

cDNAs were synthesized using a cDNA synthesis kit (TaKaRa). H_2_O was added up to 6 μL to the solution containing 1000 ng of RNA and 1 μL of random primers, and heated to 70 °C for 10 min. The solution was rapidly placed on ice for 2 min. Up to 2 μL of MLV buffer, 0.5 μL of 10 mM each dNTP, 0.25 μL of RRI, and 0.25 μL of M-MLV were added. The reaction mixture was heated to 30 °C for 10 min, 42 °C for 1 h, and to 70 °C for 15 min.

RT-qPCR was carried out using SYBR Premix Ex TaqII (TaKaRa). The reaction solution contained 10 μL of SYBR, 0.8 μL of primer pair, 1 μL of cDNA, and 8.2 μL of H_2_O. Four target genes were chosen for expression analysis with 16S rRNA as the endogenous control. The primers, shown in Table 5, were designed using the sequences of the *Xoo* genome. The PCR cycle consisted of the following steps: 30 s at 95 °C and 40 cycles of 20 s at 95 °C and 30 s at 59 °C. After each run, a dissociation curve was designed to confirm specificity of the product and avoid production of primer dimers. Relative amounts of amplification products were calculated with the comparative 2^−∆∆Ct^ method. A total of three independent biological replicates were used for each treatment.

**Table 5:** Groups of RT-qPCR primers used to amplify gene-specific regions.

Gene Name	GenBank Acc. No.	Primers (5′–3′)
*gumB* [32]	3265197	Forward: CTGACCGAAATCGAGAAGGGCACCAATC
		Reverse: GCGCCACACCATCACAAGAGGAGTCAGTTC
*gumG*	AF147035	Forward: GTCACAATGCTTGCTTACA
		Reverse: ATGGCGATGAAGAACAAC
*gumM*	AF231924	Forward: GTTCTTCGCCAATACCAAT
		Reverse: TCTCACGACACAGATACG
*xanA*	999354	Forward: GCAGCGGCGAGATCAACT
		Reverse: AAACGCCATTCGCCAAAA

## 4. Conclusions

Sulfone derivatives containing 1,3,4-oxadiazole moieties have indicated good antibacterial activities against rice bacterial leaf blight *in vitro*. In particular, 2-(methylsulfonyl)-5-(4-fluorophenyl)-1,3,4-oxadiazole revealed the best antibacterial activity against *Xoo*, which was better than those of the commercial agents bismerthiazole and thiodiazole copper. *In vivo* antibacterial activity tests under greenhouse conditions and field trials demonstrated that 2-(methylsulfonyl)-5-(4-fluorophenyl)-1,3,4-oxadiazole was effective in reducing rice bacterial leaf blight. This study also demonstrated that 2-(methylsulfonyl)-5-(4-fluorophenyl)-1,3,4-oxadiazole could stimulate the increase in SOD and POD activities in rice, causing a marked enhancement of plant resistance against rice bacterial leaf blight. 2-(Methylsulfonyl)-5-(4-fluorophenyl)-1,3,4-oxadiazole also improved the chlorophyll content and restrained the increase in the MDA content to considerably reduce the amount of damage caused by *Xoo*. 2-(Methylsulfonyl)-5-(4-fluorophenyl)-1,3,4-oxadiazole could inhibit the production of EPS, resulting in weaker pathogenicity in the bacterial strain. It also induced the gene expression levels of *gumB*, *gumG*, *gumM*, and *xanA*, whose products are involved in the biosynthesis of the EPS.
